# MiR-148a increases glioma cell migration and invasion by downregulating GADD45A in human gliomas with *IDH1* R132H mutations

**DOI:** 10.18632/oncotarget.15867

**Published:** 2017-03-03

**Authors:** Daming Cui, Pandey Sajan, Jinlong Shi, Yiwen Shen, Ke Wang, Xianyu Deng, Lin Zhou, Pingping Hu, Liang Gao

**Affiliations:** ^1^ Department of Neurosurgery, Shanghai Tenth People’s Hospital, Tongji University School of Medicine, Shanghai 200072, People’s Republic of China; ^2^ Department of Neurosurgery, Affiliated Hospital of Nantong University, Nantong 226001, Jiangsu Province, People’s Republic of China; ^3^ Department of Neurosurgery, Huashan Hospital, Fudan University, Shanghai 200070, People’s Republic of China

**Keywords:** GADD45A, miR-148a, β-catenin, migration, invasion

## Abstract

High-grade gliomas are severe tumors with poor prognosis. An R132H mutation in the isocitrate dehydrogenase (*IDH1*) gene prolongs the life of glioma patients. In this study, we investigated which genes are differentially regulated in IDH1 wild type (IDH1*^WT^*) or IDH1 R132H mutation (IDH1*^R132H^*) glioblastoma cells. Growth arrest and DNA-damage-inducible protein (GADD45A) was downregulated and microRNA 148a (miR-148a) was upregulated in in IDH1*^R132H^* human glioblastomas tissues. The relationship between GADD45A and miR-148a is unknown. *In vitro* experiments showed that *GADD45A* negatively regulates IDH1^R132H^ glioma cell proliferation, migration, and invasion, and neurosphere formation in IDH1*^R132H^* glioblastoma stem cells (GSC). In addition, a human orthotopic xenograft mouse model showed that *GADD45A* reduced tumorigenesis *in vivo*. Our findings demonstrated that miR-148a promotes glioma cell invasion and tumorigenesis by downregulating GADD45A. Our findings provide novel insights into how GADD45A is downregulated by miR-148a in IDH1*^R132H^* glioma and may help to identify therapeutic targets for the effective treatment of high-grade glioma.

## INTRODUCTION

Gliomas are the most prevalent primary brain tumors and are highly aggressive and malignant [1]. Higher-grade gliomas are more severe and have been associated with poor prognosis in human patients [2]. The genetics, etiology, and treatment of gliomas have been well investigated [1, 3, 4], but targeted treatments are still needed to improve the prognosis [5]. Understanding the pathways that promote or reduce survival will promote the development of essential new treatments.

Isocitrate dehydrogenase (*IDH1*) mutations are frequent in glioma patients [6, 7]. The R132H mutation (IDH1*^R132H^*) is the most common [7, 8]. This mutation significantly improves the prognosis of patients with glioblastoma [9]. We recently showed that the *IDH1* R132H mutation reduces the proliferation and invasion of human glioblastoma cells, indicating a tumor suppressor function. These effects were mediated by negatively regulating β-catenin signaling [10]. Wnt/β-catenin signaling is mediated by microRNAs (miRNAs), which regulate cancer-related genes. They have been used to classify [11] and detect [12] different cancers, and may represent therapeutic targets through oncogenic and tumor suppressor functions [13, 14].

To better understand the function of the *IDH1* R132H mutation, we investigated the effect of this mutation on gene expression in glioma tissues. MiR-148a expression was enhanced and growth arrest and DNA-damage-inducible protein (GADD45A) expression was reduced in human IDH1*^R132H^* gliomas. MicroRNA 148a (MiR-148a) is aberrantly expressed in cancer tissues [15]. It is highly expressed in glioblastoma tissues [16] and regulates glioma development and progression [17, 18]. Upregulation of miR-148a promotes malignancy and reduces patient survival [16, 19]. In contrast, GADD45A reduces cancer progression by promoting apoptosis and cell-cycle arrest [20–24].

In contrast to previous reports that *IDH1* R132H mutations promote survival, we confirmed that miR-148a increased cell migration and invasion by downregulating GADD45A in IDH1*^R132H^* glioblastomas. Our findings provide a deeper insight into how miR-148a is increased in IDH1*^R132H^* gliomas.

## RESULTS

### GADD45A and miR-148a expression in IDH1*^WT^* and IDH1*^R132H^* glioma tissues

**Figure 1:**
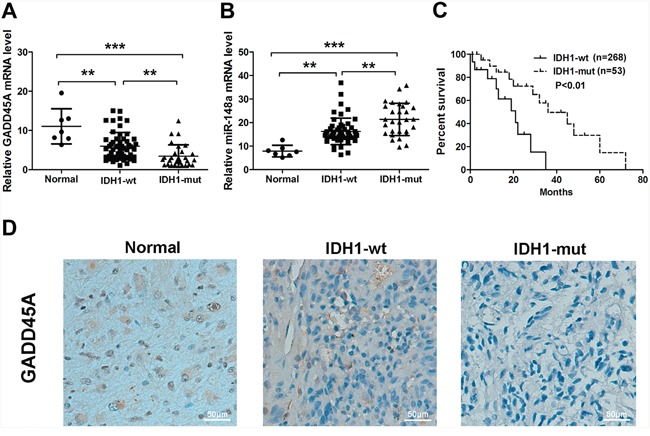
GADD45A and miR-148a expression in normal tissues and IDH1^*WT*^ or IDH1^*R132H*^ glioma tissues **(A–B)** qRT-PCR analysis of *GADD45A* and miR-148a mRNA expression in the three tissue types. **(C)** Kaplan-Meier analysis of the relationship between IDH1*^R132H^* (n=53) and IDH1*^WT^* (n=268) with patient survival in glioma patients (P<0.01, Log-rank test). **(D)** GADD45A immunostaining revealed lower protein expression in IDH1*^R132H^* glioma tissues compared with normal tissues and IDH1*^WT^* gliomas. Magnification: ×200. **P<0.01, ***P<0.001.

To investigate which genes are differentially expressed in *IDH1* wild type (IDH1*^WT^*) and IDH1*^R132H^* glioma cells, we performed microarray analysis (Supplementary Figure 1). GADD45A was significantly downregulated in IDH1*^R132H^* gilomas cells compared with IDH1*^WT^* cells (Supplementary gene-list.xls). Clinicopathological characteristics of 81 gliomas patients are presented in Table 1. Patients were divided into two groups based on the intensity of GADD45A immunostaining. Glioma tissue samples included 30 WHO grade I–II (15 with IDH1*^R132H^*), 26 WHO grade III (12 with IDH1*^R132H^*), and 25 WHO grade IV tumors (two with IDH1*^R132H^*). GADD45A was significantly downregulated in IDH1*^R132H^* tumors compared with IDH1*^WT^*. To confirm differential expression of *GADD45A* and miR-148a, we measured *GADD45A* and miR-148a mRNA levels in the same human glioma tissues using qRT-PCR. *GADD45A* expression was higher in normal tissues compared with glioma tissues (Figure 1A) and was lower in IDH1*^R132H^* glioma tissue than IDH1*^WT^* glioma (P<0.01). In contrast, miR-148a expression was lower in normal tissues compared with glioma tissues (Figure 1B) and was higher in IDH1*^R132H^* glioma tissue than IDH1*^WT^* gliomas (P<0.01).

**Table 1:** GADD45A staining and clinicopathological characteristics of 81 gliomas patients

*Age (years)*	GADD45A expression		Total	P-value
	Low	High		
	Negative (%)	Positive (%)		0.383
<50	30 (69.77)	13 (30.23)	43	
≥50	23 (60.53)	15 (39.47)	38	
***Gender***				0.071
Male	31 (58.49)	22 (41.51)	53	
Female	22 (78.57)	6 (21.43)	28	
***WHO grade***				0.025*
Low-grade (I–II)	15 (50.00)	15 (50.00)	30	
High-grade (III–IV)	38 (74.51)	13 (25.49)	51	
***IDH1***				0.049*
Wild type	30 (57.69)	22 (42.31)	52	
Mutation	23 (79.31)	6 (20.69)	29	

*P<0.05, Chi-square test.

We analyzed data in the Cancer Genome Atlas (TCGA) to investigate the correlation between IDH1*^R132H^* and IDH1*^WT^* patient survival. Kaplan-Meier analysis showed that IDH1*^R132H^* correlated positively with overall survival (P<0.01, Log-rank test; Figure 1C).

We examined GADD45A protein expression in normal and glioma tissues by immunohistochemistry. GADD45A staining appeared to be stronger in normal tissues than glioma tissues. In addition, staining was stronger in IDH1*^WT^* than IDH1*^R132H^* glioma tissue (Figure 1D).

### The *IDH1* R132H mutation decreases GADD45A while increases miR148a expression in glioblastoma cell lines

**Figure 2:**
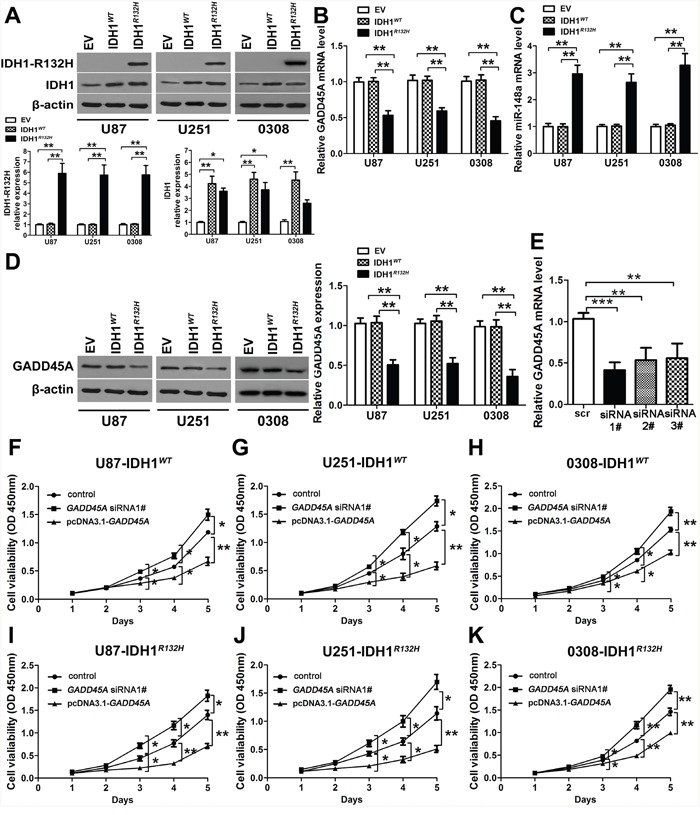
GADD45A inhibits cell proliferation *in vitro* **(A)** Western blot analysis of IDH1*^WT^* and IDH1*^R132H^* protein expression in U87 and U251 glioblastoma cell lines and GSC 0308 cells after stable transfection with empty vector (EV), *IDH1^WT^*, and *IDH1^R132H^*. **(B–C)** qRT-PCR analysis of *GADD45A* and miR-148a expression in U87 and U251 glioblastoma cell lines and GSC 0308 cells stably transfected with EV, *IDH1^WT^*, and *IDH1^R132H^*. **(D)** Western blot analysis of GADD45A expression in U87, U251 glioblastoma cell lines and GSC 0308 stably transfected with EV, *IDH1^WT^*, or *IDH1^R132H^*. **(E)** *GADD45A* was silenced in U87 cells by three different siRNAs (siRNA#1–3) as shown by qRT-PCR. **(F–K)** The effect of *GADD45A* knockdown and overexpression on cell viability in IHD1*^WT^* or IDH1*^R132H^* U87, U251, and GSC 0308 cells. Non-transfected IHD1*^WT^* or IDH1*^R132H^* U87, U251 and 0308 cells were used as controls. *P<0.05, **P<0.01, ***P<0.001.

We stably expressed IDH1*^WT^* or IDH1*^R132H^* in U87 cells, U251 cells, and the glioblastoma stem cell (GSC) line 0308 by lentiviral infection. Expression of IDH1*^WT^* or IDH1*^R132H^* protein was confirmed in both cell lines by western blotting. Cells infected with lentiviral particles carrying the empty vector (EV) were used as controls (Figure 2A). IDH1*^R132H^*protein was only detected in the IDH1*^R132H^* cell lines, and was overexpressed by 6-fold compared with EV or IDH1*^WT^* cell lines, while IDH1*^WT^* protein was detected in IDH1*^WT^* and IDH1*^R132H^* glioblastoma cells and GSCs and was overexpressed 4-fold over endogenous IDH1 (Figure 2A), these were in agreement with previous reports [10, 25]. *GADD45A* mRNA expression was reduced (Figure 2B) and miR-148a expression was increased in IDH1*^R132H^* cells (Figure 2C). However, expression was not different in EV and IDH1*^WT^* cells. We confirmed a reduction of GADD45A expression on the protein level in IDH1*^R132H^* cells compared with EV and IDH1*^WT^* cells by western blotting (Figure 2D).

### GADD45A expression inhibits glioblastoma cell proliferation *in vitro*

We knocked down *GADD45A* expression in glioblastoma cells using three different siRNAs (Figure 2E). *GADD45A* was overexpressed by introducing a pcDNA3.1-*GADD45A* plasmid (Figure 2F–2K). After *GADD45A* knockdown or overexpression, we measured cell proliferation of IDH1*^WT^* or IDH1*^R132H^* U87, U251, and 0308 cells. *GADD45A* knockdown increased cell proliferation while *GADD45A* overexpression reduced cell proliferation in IDH1*^WT^* and IDH1*^R132H^* cells (Figure 2F–2K). Taken together, these findings indicated that GADD45A inhibits glioma cell and GSC proliferation *in vitro*.

### GADD45A expression suppresses glioblastoma tumor growth *in vivo*

**Figure 3:**
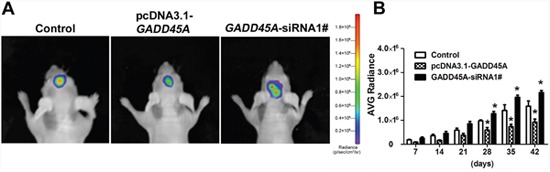
GADD45A inhibits tumor growth *in vitro* **(A)** Implanted U87 MG-luc2 cells stably expressing pcDNA3.1-*GADD45A* or *GADD45A*-siRNA1# are visible in mouse brains as red–blue signals. Red indicates the highest Bioluminescence imaging (BLI) signal intensity. The BLI signal intensity increased from day 42, indicating progressive growth of the glioblastoma xenograft. **(B)** Average IVIS values of mice, data are expressed as mean ± SD. *P<0.05.

To investigate the influence of *GADD45A* expression on tumor growth *in vivo*, we injected mice with IDH1*^R132H^*U87-Luc2 cells that stably expressed either pcDNA3.1-*GADD45A* or *GADD45A*-siRNA and monitored tumor growth. We observed pcDNA3.1-*GADD45A* or *GADD45A*-siRNA glioblastoma cells in mouse brains *in vivo* by bioluminescence imaging (BLI) (Figure 3A). BLI showed increasing radiance values corresponding to increasing tumor growth higher in GADD45A-siRNA tumors compared with control, while pcDNA3.1-GADD45A showed a lower radiance than control (Figure 3). Taken together, these findings suggested that GADD45A inhibits glioma tumorigenesis *in vivo*.

### MiR-148a targets GADD45A

**Figure 4:**
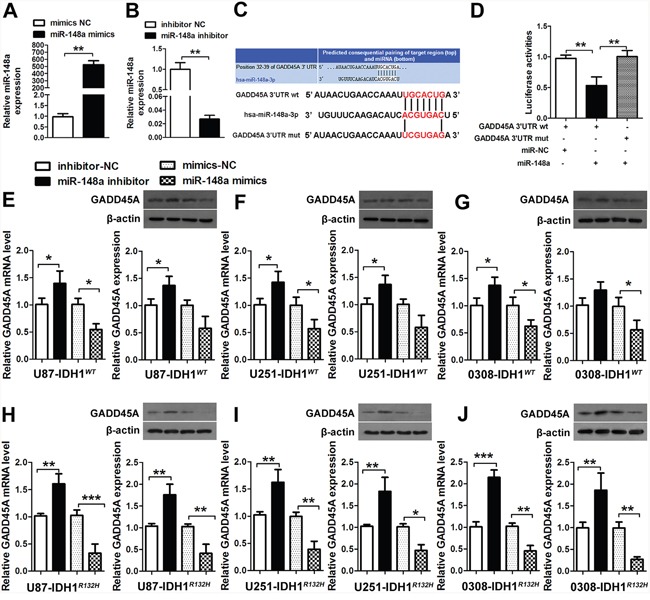
miR-148a binds GADD45A **(A-B)** U87 cells were transfected with miR-148a mimics, mimics-NC, miR-148a inhibitor, or inhibitor-NC for 48 h. The miR-148a levels were determined by qRT-PCR. **(C)** The putative miR-148a binding sites in the *GADD45A* sequence. **(D)** The luciferase reporter plasmid containing wild type or mutant *GADD45A* 3′-UTR was co-transfected into HEK-293 T cells with miR-148a mimics or miR-148a mimics-NC. Luciferase activity was determined 48 h after transfection and was normalized to Renilla activity. **(E–G)** GADD45A expression in IDH1*^WT^* U87, U251, and GSC 0308 cells transfected with miR-148a inhibitor/ miR-148a inhibitor-NC, or miR-148a mimics/miR-148a mimics-NC for 48 h was measured by qRT-PCR and western blotting. **(H–J)** qRT-PCR analysis of *GADD45A* mrna and western blot analysis of GADD45A expression in IDH1*^R132H^* U87, U251, and 0308 cells transfected with miR-148a inhibitor/miR-148a inhibitor-NC or miR-148a mimics/miR-148a mimics-NC for 48 h. β-actin was used as a control. *P<0.05, **P<0.01, ***P<0.001.

The expression patterns of GADD45A and miR-148a were opposite in human glioma tissues. To investigate whether *GADD45A* expression is downregulated by miR-148a, we transfected U87 cells with miR-148a mimics, mimics-NC, miR-148a inhibitor, or inhibitor-NC for 48 h. Upregulation of miR-148a expression by miR-148a mimics significantly increased miR-148a levels and miR-148a inhibitors significantly reduced miR-148a expression (Figure 4A–4B).

To investigate the relationship between *GADD45A* and miR-148a, we searched for a putative miRNA binding site in the *GADD45A* sequence (Figure 4C). We used luciferase reporter assays to confirm regulation of *GADD45A* expression by miR-148a. The luciferase reporter plasmid containing wild type or mutant *GADD45A* 3′-UTR sequences was co-transfected into HEK-293 cells with miR-148a mimics or miR-NC (Figure 4D). Luciferase activity was measured 48 h after transfection and was normalized to Renilla activity. Luciferase activity of wild type *GADD45A* was significantly reduced when co-transfected with miR-148a mimics compared with miR-NC, while the activity of mutant *GADD45A* was not altered. These results suggested that miR-148a negatively regulates *GADD45A* expression by binding to a specific sequence in the 3′-UTR.

MiR-148a mimics or miR-148a inhibitors were transfected into IDH1*^WT^* and IDH1*^R132H^* U87, U251, and 0308 cells. In IDH1*^WT^* cells, miR-148a inhibitors increased GADD45A mRNA and protein expression and miR-148a mimics reduced GADD45A expression (Figure 4E–4G). In IDH1*^R132H^* glioblastoma cells, miR-148a inhibitors significantly increased and miR-148a mimics significantly decreased GADD45A expression (Figure 4H–4J). These findings provided further support that miR-148a is a direct target of GADD45A in IDH1*^R132H^* glioma cells and GSC.

### GADD45A overexpression inhibits the expression of β-catenin and MMP, and the epithelial-mesenchymal transition (EMT) in glioblastoma cells

**Figure 5:**
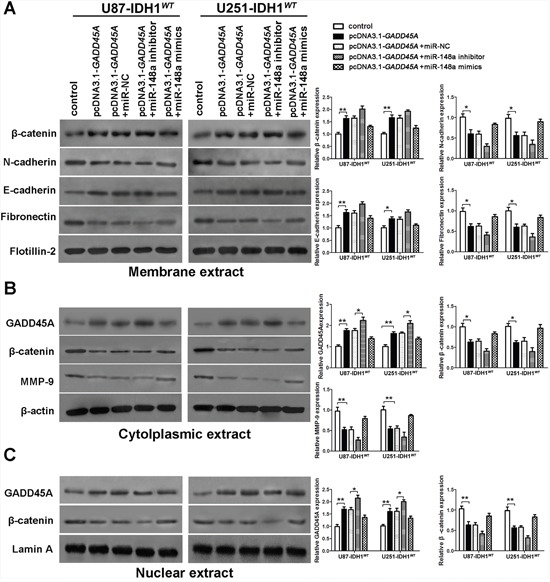
miR-148a partly stimulates β-catenin, MMP-9, and the epithelial-mesenchymal transition by downregulating GADD45A in IDH1^WT^ glioblastoma cells **(A)** Western blot analysis of β-catenin, N-cadherin, E-cadherin, and fibronectin expression in membrane extracts. Flotillin-2 was used as a membrane marker. **(B)** Western blot analysis of GADD45A, β-catenin, and MMP-9 expression in cytoplasmic extracts. β-actin was used as a control. **(C)** Western blot analysis of β-catenin and GADD45A expression in nuclear extracts. Lamin A was used as a control. All extracts were prepared from IDH1*^WT^* U87 and U251 glioblastoma cells. *P<0.05, **P<0.01.

**Figure 6:**
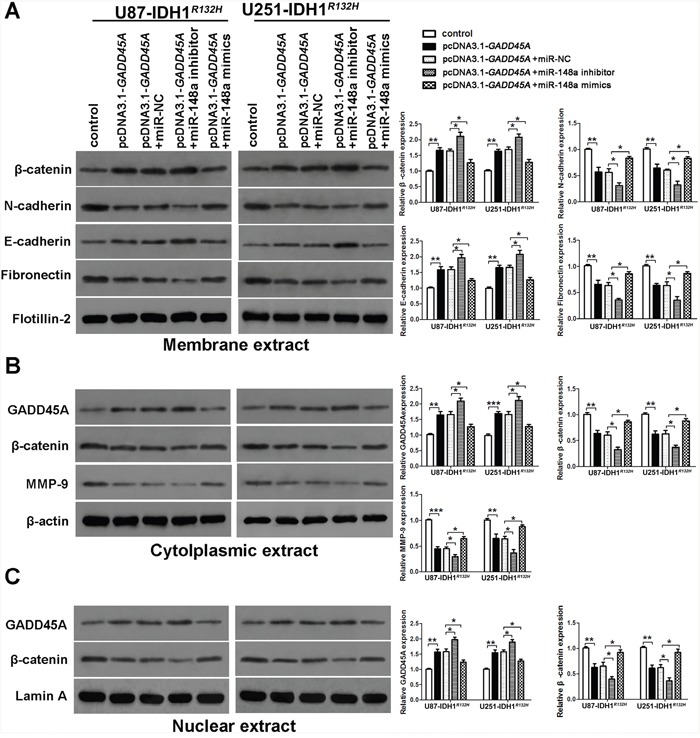
miR-148a stimulates β-catenin, MMP-9, and the epithelial-mesenchymal transition by inhibiting GADD45A in IDH1^*R132H*^ glioblastoma cells **(A)** Western blot analysis of β-catenin, N-cadherin, E-cadherin, and fibronectin expression in membrane extracts. Flotillin-2 was used as a membrane marker. **(B)** Western blot analysis of GADD45A, β-catenin, and MMP-9 expression in cytoplasmic extracts. β-actin was used as a control. **(C)** Western blot analysis of β-catenin and GADD45A expression in nuclear extracts. Lamin A was used as a control. All extracts were prepared from IDH1*^R132H^* U87 and U251 glioblastoma cells. *P<0.05, **P<0.01, ***P<0.001.

To investigate whether GADD45A inhibits expression of β-catenin, MMP-9, and EMT markers, we performed western blotting on total protein extracts from IDH1*^WT^* or IDH1*^R132H^* glioblastoma cell extracts after *GADD45A* overexpression. We prepared membrane, cytoplasmic, and nuclear extracts from both cell types for western blotting (Figure 5A–5C and Figure 6A–6C, respectively). *GADD45A* overexpression inhibited β-catenin, MMP-9, N-cadherin, and fibronectin expression in cytoplasmic and nuclear (β-catenin only) extracts. In contrast, *GADD45A* overexpression increased E-cadherin expression in IDH1*^WT^* and IDH1*^R132H^* glioblastoma cells (Figure 5A–5C and Figure 6A–6C). These findings suggested that GADD45A inhibits the EMT in glioblastoma cells.

### MiR-148a stimulates β-catenin, MMP-9, and EMT marker expression by downregulating GADD45A

To determine the effect of miR-148a on GADD45A-mediated control of β-catenin, MMP-9, and EMT marker expression, we upregulated or downregulated miR-148a in IDH1*^WT^* or IDH1*^R132H^* glioblastoma cells overexpressing *GADD45A* (Figure 5A–5C and Figure 6A–6C). Upregulation of miR-148a only significantly increases β-catenin, MMP-9, and EMT marker expression in IDH1*^R132H^* glioblastoma cells by downregulating GADD45A. These findings suggested that miR-148a inhibits GADD45A in IDH1*^R132H^* gliomas cells.

### MiR-148a increases glioblastoma cell migration and invasion, and stimulates the cellular distribution of β-catenin

**Figure 7:**
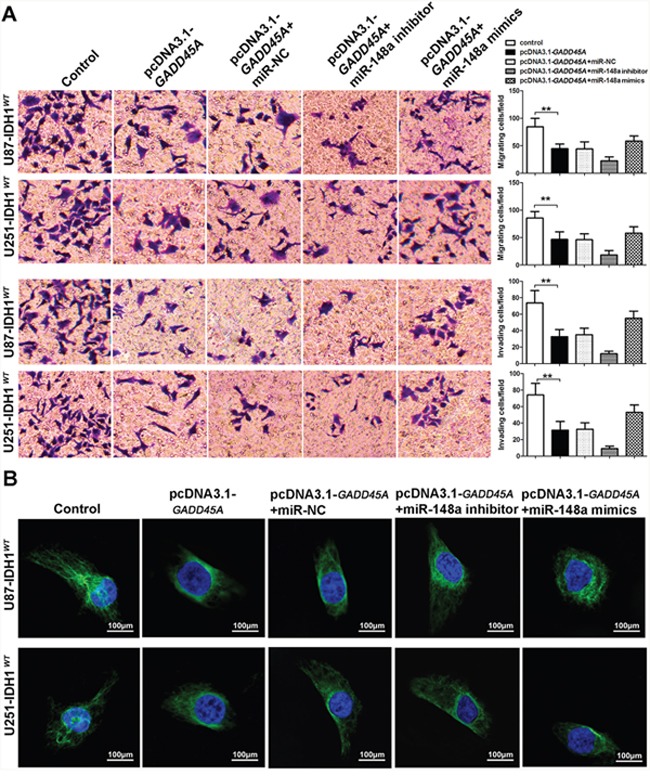
miR-148a partly increases cell migration and invasion and β-catenin distribution by downregulating GADD45A in IDH1^WT^ glioblastoma cells **(A)** Migration and invasion of IDH1*^WT^* U87 and U251 glioblastoma cells were measured using transwell assays. **(B)** Immunofluorescence staining of β-catenin in IDH1*^WT^* U87 and U251 cells. **P<0.01.

**Figure 8:**
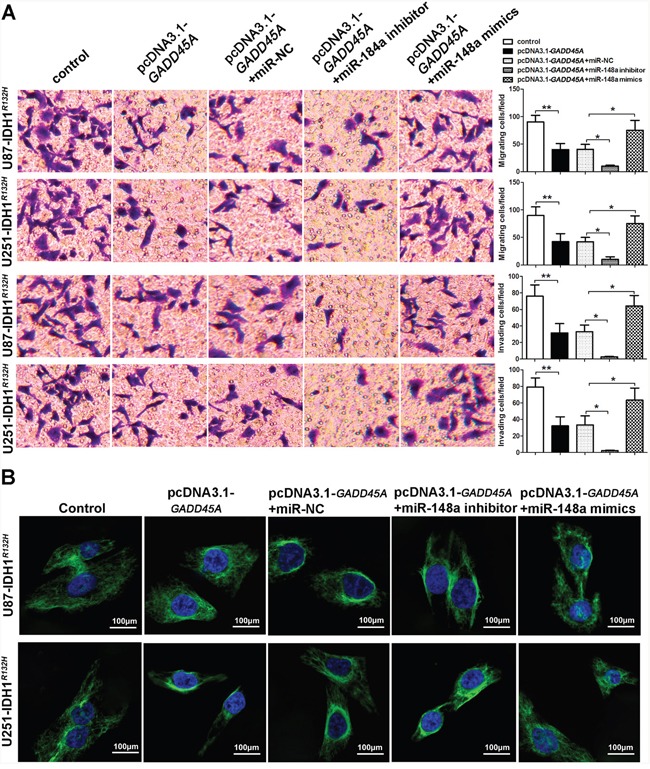
miR-148a increases cell migration and invasion and β-catenin distribution by inhibiting GADD45A in IDH1^*R132H*^ U87 and U251 cells **(A)** Migration and invasion of IDH1*^R132H^* U87 and U251 glioblastoma cells were measured using transwell assays. **(B)** Immunofluorescence staining of β-catenin in IDH1*^R132H^* U87 and U251 cells. *GADD45A* overexpression removed β-catenin from the nucleus and this effect was antagonized by miR-148a. *P<0.05, **P<0.01.

To investigate the effect of miR-148a on glioblastoma cell migration and invasion, we performed transwell assays. *GADD45A* overexpression reduced the migration and invasive ability of IDH1*^WT^* and IDH1*^R132H^* glioblastoma cells (Figure 7A and Figure 8A). The effect of GADD45A on glioblastoma cell proliferation was inhibited by miR-148a inhibitors and increased by miR-148a mimics in IDH1*^WT^* and IDH1*^R132H^* glioblastoma cells. However, this effect was only significant in IDH1*^R132H^* cells. This indicates that miR-148a significantly inhibits GADD45A to increase the migration and invasion of IDH1*^R132H^* glioma cells.

We observed that miR-148a controls β-catenin expression by targeting *GADD45A*. To explore the effect of miR-148a and GADD45A on the cellular distribution of β-catenin, we performed immunofluorescence staining experiments. β-catenin was distributed evenly throughout the nucleus and cytoplasm of control cells (Figure 7B and Figure 8B). *GADD45A* overexpression removed β-catenin from the nucleus and increased staining at the membrane. Inhibition of miR-148a enhanced this effect, while only upregulation of miR-148a increased β-catenin staining in the nucleus in IDH1*^R132H^* cells (Figure 8B). Taken together, these findings show that miR-148a stimulates the cellular distribution of β-catenin by inhibiting *GADD45A* in IDH1*^R132H^* glioma cells.

### MiR-148a stimulates GSC neurosphere formation by inhibiting GADD45A

**Figure 9:**
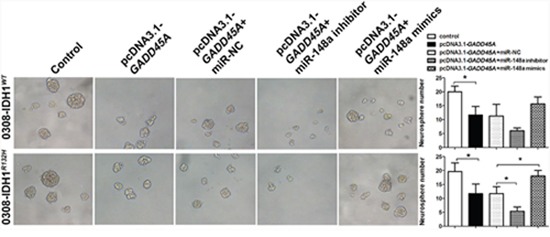
MiR-148a increases GSC neurosphere formation by downregulating GADD45A in IDH1^WT^ and IDH1^*R132H*^ cells GSCs were transfected with pcDNA3.1-*GADD45A* without or with miR-148a inhibitor, miR-148a mimics, or controls and neurosphere formation was measured *P<0.05, **P<0.01.

MiR-148a has been implicated in GSC neurosphere self-renewal [16]. In the present study, *GADD45A* overexpression significantly reduced the size and number of IDH1*^WT^* or IDH1*^R132H^* GSC 0308 neurospheres (Figure 9). We showed that miR-148a partly reverses GADD45A-inhibited effects in IDH1*^WT^* GSC 0308 cells. In contrast, miR-148a significantly promotes the self-renewal ability of IDH1*^R132H^* GSC 0308 cells by inhibiting GADD45A expression. These results indicated that miR-148a significantly inhibits GADD45A expression in IDH1*^R132H^* GSCs.

### GADD45A mediates the effects of miR-148a in IDH1*^R132H^* glioblastoma cells and stem cells

**Figure 10:**
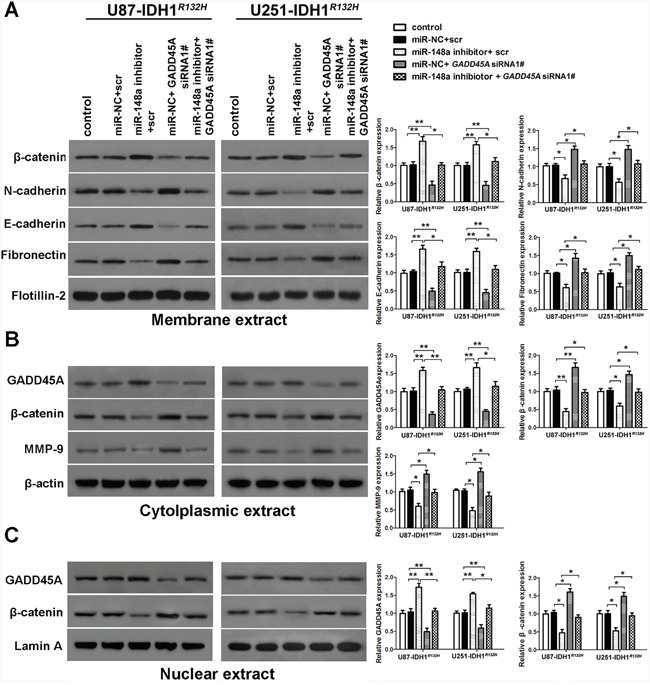
GADD45A inhibits the effects of miR-148a on β-catenin, MMP-9, and EMT marker expression in IDH1^*R132H*^ glioblastoma cells **(A)** Western blot analysis of β-catenin, N-cadherin, E-cadherin, and fibronectin expression in membrane extracts. Flotillin-2 was used as a membrane marker. **(B)** Western blot analysis of GADD45A, β-catenin, and MMP-9 expression in cytoplasmic extracts. β-actin was used as a control. **(C)** Western blots analysis of β-catenin and GADD45A expression in nuclear extracts. Lamin A was used as a control. IDH1*^R132H^* U87 and U251 cells were transfected with miR-148a inhibitor before transfection with *GADD45A* siRNA1# or scrambled controls. *GADD45A* siRNA1# rescued the effects of miR-148a inhibition on protein expression. *P<0.05, **P<0.01.

**Figure 11:**
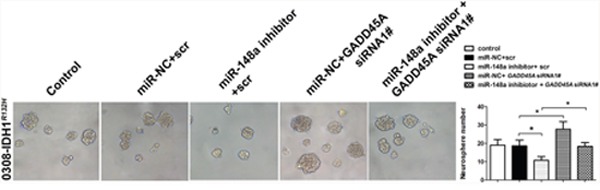
GADD45A inhibits the effects of miR-148a on IDH1^*R132H*^ GSC neurosphere formation GSCs were transfected with miR-148a inhibitor before transfection with *GADD45A* siRNA1# or scrambled controls. *GADD45A* siRNA1# rescued the effects of miR-148a inhibition on neurosphere formation. *P<0.05, **P<0.01.

We investigated whether miR-148a-stimulated oncogenesis is inhibited by GADD45A. IDH1*^R132H^* glioblastoma cells were transfected with scrambled or *GADD45A* siRNA together with miR-148a inhibitors. Then, the expression of β-catenin, EMT markers, and MMP-9 was measured. Inhibition of miR-148a significantly reduced β-catenin, EMT marker, and MMP-9 expression in IDH1*^R132H^* glioblastoma cells and these effects were prevented by *GADD45A* knockdown (Figure 10). MiR-148a inhibition increased the formation of IDH1*^R132H^* GSC neurospheres and this effect was eliminated by *GADD45A* knockdown (Figure 11). Taken together, these findings show that GADD45A suppresses the effects of miR-148a in I IDH1*^R132H^* gliomas.

## DISCUSSION

The *IDH1* R132H mutation promotes the survival of glioma patients. Understanding the underlying mechanisms may uncover novel treatments for the disease. In the current study, we searched for novel genes that were differentially expressed in *IDH1* R132H glioma cells and revealed a downregulation of *GADD45A*. GADD45A reduced IDH1*^R132H^* glioblastoma cell proliferation and tumor growth. This was in agreement with previous reports that GADD45A reduces cancer progression [20] by promoting apoptosis and cell-cycle arrest [21–24]. In addition, we found that miR-148a was upregulated in IDH1*^R132H^* glioma tissue and inhibited the tumor suppressor function of GADD45A by downregulating its expression.

We have previously shown that the *IDH1* R132H mutation reduces glioblastoma progression by inhibiting Wnt/β-catenin signaling [10]. β-catenin signaling plays several roles in cancer progression [26–28]. Importantly, when β-catenin is inhibited or prevented from translocating into the nucleus, apoptosis and cell-cycle arrest is promoted and cell proliferation is reduced [29–31].

GADD45A prevents the EMT by controlling the expression of matrix metalloproteinases (MMPs) [20] and β-catenin distribution within cancer cells [32]. In this study, we confirmed that GADD45A reduced the expression of β-catenin and MMP-9 in IDH1*^R132H^* glioblastoma cells, providing a deeper insight into how β-catenin is regulated in these tumors [10]. GADD45A-mediated regulation of the EMT was inhibited by miR-148a. This is in agreement with previous findings that miR-148a inhibits metastasis by blocking the EMT [33, 34].

*GADD45A* overexpression promoted the redistribution of β-catenin from the nucleus and cytoplasm to the membrane in IDH1*^R132H^* glioblastoma cells [10]. Furthermore, GADD45A increased E-cadherin expression at the membrane and inhibited glioblastoma cell invasion. This supports previous findings that GADD45A induces β-catenin translocation to the cell membrane to stabilize focal adhesions and promote contact inhibition, thereby preventing tumorigenesis [32].

MiR-148a was reported to be downregulated in IDH1*^R132H^* gliomas due to miR148a promoter hypermethylation [35]. This contradicts our finding that miR-148a is upregulated in IDH1 *^R132H^* glioma tissues. Further support for our findings comes from another study showing that miR-148 is upregulated in >500 human glioblastoma tissues [16]. The authors showed that miR-148a exerted an oncogenic effect and reduced patient survival by targeting the EGFR regulator MIG6 and the apoptosis regulator BIM [16]. In the present study, we confirmed the oncogenic potential of miR-148a and identified a novel miR-148a target, *GADD45A*.

In conclusion, we have demonstrated that the tumor suppressor gene *GADD45A* and the miR-148a are differentially expressed in IDH1*^R132H^* gliomas. MiR-148a promotes malignancy in these gliomas by inhibiting the tumor suppressor function of GADD45A.

## MATERIALS AND METHODS

### Human tissue samples

Tumor tissues were collected from glioma patients admitted to the Shanghai Tenth People’s Hospital and control tissues were obtained from healthy individuals as previously described [10]. Tumor tissues were classified according to the 2007 WHO classification. Brain tissues were collected from seven healthy individuals and 81 glioma patients and flash frozen. All participants provided written informed consent according to the ethical guidelines of the Shanghai Tenth People’s Hospital ethics committee (Tongji University, Shanghai, China).

### Microarray analysis

For microarray-based comparison of gene expression, total RNA was isolated from IDH1*^WT^* or IDH1*^R132H^* U87 glioblastoma cells. Samples were processed by Invitrogen Biotechnology (Shanghai, China). RNA quality was analyzed, and then the transcripts were hybridized to Affymetrix GeneChip Human Exon 1.0 ST Arrays. The arrays were scanned using an Affymetrix GeneChip scanner 3000 7G system. Differentially expressed genes were identified using the TwoClassDif method.

### Database analysis

We obtained whole genome mRNA microarray data and clinical information for gliomas as a discovery set from the Chinese Glioma Genome Atlas database (http://www.cgga.org.cn). In addition, we analyzed 268 IDH1*^WT^* (n=268) and IDH1*^R132H^* (n=53) gliomas from the TCGA database as previously described [36].

### Cell culture

The human glioma cell lines U87 and U251 were obtained from the Chinese Academy of Sciences Cell Bank (Shanghai, China). The GSC 0308 cell line was obtained from the American Type Culture Collection (ATCC, Manassas, VA, USA). Glioma cells were cultured in Dulbecco’s modified Eagle’s medium (Gibco, Grand Island, NY, USA) supplemented with 10% fetal bovine serum (FBS) (Gibco), 2 mM glutamine and 100 ug/ml penicillin/streptomycin, at 37°C and 5% CO_2_, as previously described [10]. GSC were cultured in DMEM/F12 medium supplemented with 20% BIT, 100 ug/ml penicillin/streptomycin, 20 ng/ml epidermal growth factor (EGF), and 20 ng/ml basic fibroblast growth factor. U87MG-luc2 (U87-luc2), a human glioblastoma cell line was purchased from Caliper Life Sciences (Hopkinton, USA). The cells were grown in Minimum Essential Medium (Invitrogen) with 10% FBS and 100 ug/ml penicillin/streptomycin at 37°C and 5% CO_2_.according to previously study [37].

### RNA isolation and real-time quantitative RT PCR (qRT-PCR)

Total RNA was extracted from fresh-frozen tissues or cells using Trizol reagent (Invitrogen, Carlsbad, CA, USA) and miRNA molecules were purified using the mirVana miRNA isolation kit (Ambion, Austin, USA) as previously described [38]. RNA was reverse transcribed using the PrimeScript RT reagent kit (Takara, Dalian, China). qRT-PCR was performed using SYBR Premix Ex Taq II (Takara) on an ABI Prism 7500 PCR system (Applied Biosystems, USA). Data were normalized to β-actin. Mature miR-148a was analyzed by qRT-PCR using the TaqMan MicroRNA Assay Kit (Applied Biosystems). The expression of mature miR-148a was determined by real-time PCR analysis following stem-loop RT and data were normalized to U6 snRNA. Relative expression was measured using the 2^−ΔΔCT^ method [39] with the following primers: *GADD45A* forward: 5′- GAGCAGAAGACCGAAAGCGAC-3′, reverse: 5′-GAATGTGGATTCGTCACCAGC-3′. MiR-148a RT-primer: 5′-GTCGTATCCAGTGCAGGGTCCGAGGTATTCGCACTGGATACGACAACAAAGTT-3′; MiR-148a PCR forward: 5′-GCTAGTGTTCTGAGACACTCCG-3′, PCR reverse: 5′-GTGCAGGGTCCGAGGT-3′. U6 RT-primer: 5′-CGCTTCACGAATTTGCGTGTCAT-3′, U6 PCR forward: 5′-GCTTCGGCAGCACATATACTAAAAT-3′, PCR reverse: 5′-CGCTTCACGAATTTGCGTGTCAT-3′.

### Immunohistochemistry

Tissues were prepared for immunohistochemical staining as previously described [10]. Paraffin-embedded sections were incubated in anti-GADD45A primary antibody (1:100 dilution; Abcam, Cambridge, USA) at 4°C overnight. After washing, sections were incubated with HRP-conjugated anti-mouse secondary antibody (1:1000 dilution; Abcam) for 1 h at 37°C. The signal was enhanced using the ABC peroxidase staining kit (ThermoFisher Scientific) and staining was detected by diaminobenzidine (DAB). Sections were counterstained with hematoxylin.

### Evaluation of immunostaining intensity

GADD45A immunostaining was analyzed using intensity and distribution measurements as previously described [40, 41]. The staining intensity was scored as 0 (no staining), 1 (weak), 2 (moderate), and 3 (strong). The staining distribution was measured as the percentage of positive tumor cells (0% to 100%). GADD45A expression was scored by multiplying the intensity with the distribution. A final score of 0 corresponded to no staining and a high score of 300 indicated 100% of cells with a staining intensity of 3. Cells were further divided according to GADD45A expression into “low or negative” (GADD45A low) and “high or positive” (GADD45A high) groups, according to a cutoff point. The cutoff point for GADD45A expression was calculated using the X-tile software program as previously described [42].

### Lentiviral constructs and cell constructs

We overexpressed IDH1*^R132H^* and IDH1*^WT^* cDNAs as previously described [10, 43]. To generate lentiviral particles, lentiviral vectors (pLenti6.3-MCS-IRES2-EGFP) (Invitrogen) were co-transfected with packaging vectors pLP1, pLP2, and pLP/VSVG into 293T cells using Lipofectamine 2000 (Life Technologies, Carlsbad, CA, USA) according to the manufacturer’s instructions. Supernatants were collected 48 h and 72 h after transfection and concentrated by ultracentrifugation. The virus titer was determined and lentiviral particles were used to infect the target cells. Infected cells were selected by 6 μg/ml blasticidin (Invitrogen) for 2 weeks to generate stable-transfected cell lines.

### Plasmid construction

*GADD45A* expression was knocked down using three short interfering RNAs (siRNA) as follows: *GADD45A*-siRNA1# (5′-ATAAGTTGACTTAAGGCAGGA-3′), GADD45A-siRNA2# (5′-CATTGATCCATGTAGCGA CTT-3′), GADD45A-siRNA3# (5′-AACCCATTGATC CATGTAGCG-3′) and a scramble negative siRNA (scr) (5′-GACCTGTACGCCAACACAGTG-3′). The siRNAs were chemically synthesized (Genechem, Shanghai, China) and subcloned into a psilencer 4.1 vector (Invitrogen). Transfected cells were selected using puromycin to generate stable cell lines. To overexpress *GADD45A*, the following primers were used for amplification: forward: 5′-GCGGGTACCATGACTTTGGAGGAATTCTC-3′ and reverse: 5′-GGCCTCGAGTCACCGTTCAGGGAGATTAA-3′. The *GADD45A* cDNA product was cloned into the mammalian expression pcDNA3.1(+) vector (Invitrogen) at *Kpn*I and *Xho*I restriction sites (TaKaRa, Dalian, China). Stable colonies were selected using Geneticin (G418).

### Plasmid transfection

Plasmid DNA was extracted using a DNA Midiprep kit (Qiagen, Hilden, Germany). *GADD45A* siRNAs and scr were purchased from GeneChem (Shanghai, China). *GADD45A* siRNA1#, pcDNA3.1-*gadd45a*, miR-148a mimics or miR-148a inhibitor (GenePharma, Shanghai, China) were transfected into U87, U251, and GSC 0308 cells that stably expressed IDH1*^WT^* or IDH1*^R132H^* using Lipofectamine 2000 (Invitrogen). Forty-eight hours after transfection, cells were harvested for qRT-PCR or western blotting.

### Western blotting

To measure protein expression in glioma cells, we prepared cellular extracts using the NE-PER® nuclear, cytoplasmic, and membrane extraction kit (Thermo Scientific, Waltham, MA, USA), according to the manufacturer’s instructions. Protein extracts were separated by SDS-PAGE on a 12% polyacrylamide gel, and then transferred to a PVDF membrane (Millipore, Bedford, MA, USA). Membranes were blocked at room temperature with 5% non-fat milk before incubating overnight at 4°C with the following primary antibodies: anti-IDH1 R132H mutation antibody (1:500 dilution; Dianova, Hamburg, Germany), anti-IDH1 antibody (1:500 dilution; Dianova), anti-GADD45A (1:200 dilution;Abcam, San Francisco, California, USA), anti-β-catenin (1:1,000 dilution; Abcam), anti-MMP-9 (1:1,000 dilution; Abcam), anti-E-cadherin (1:50 dilution; Abcam), anti-N-cadherin (1:1,000 dilution; Abcam), and anti-fibronectin (1:100 dilution, Abcam). Anti-β-actin (1:1,000 dilution; Abcam), anti-lamin A (1:500 dilution, Abcam) and anti-flotillin (1:500 dilution; Abcam) were used as controls. After primary antibody incubation, membranes were washed and incubated with HRP-conjugated secondary antibodies. Protein bands were visualized using the enhanced chemiluminescence (ECL) system (Pierce, Rockford, IL, USA).

### Cell proliferation assay

Cell proliferation was quantified using the Cell Counting Kit-8 (CCK-8, Dojindo, Kumamoto, Japan) according to the manufacturer’s instructions. Briefly, 2×10^3^ cells/well were seeded into 96-well plates and pre-incubated for 12 h. Then, 10 μl CCK-8 solution was added to each well and the cells were incubated for 1–5 days. Absorbance was measured at 450 nm using an Epoch Microplate Spectrophotometer (Bio Tek, Winooski, VT, USA).

### Neurosphere formation assay

GSC neurosphere formation was analyzed as previously described [16]. Briefly, IDH1*^WT^* or IDH1*^R132H^* GSCs were transfected with either miR-148a inhibitor or miR-148a mimics for 72 hours. The cells were dissociated into a single cell suspension in 1mM EDTA plus 0.5% BSA and 1,000 single cells were incubated in 6- well plate for 7 days. Neurospheres containing more than 20 cells were counted.

### Animals and orthotopic xenotransplantation

Nude male BALB/c mice (5 weeks old) were obtained from Shanghai Laboratory Animal Company (Shanghai, China). Ten mice were used per group. All experiments were approved and performed according to the guidelines of the Ethics Committee of Shanghai Tenth People’s Hospital of China and conformed to the Principles of Laboratory Animal Care (National Society for Medical Research), and National Institutes of Health guidelines. For *in vivo* imaging of glioblastoma formation, 3×10^5^ IDH1*^R132H^* U87 cells stably expressing luciferase (IDH1*^R132H^* U87-luc) that expressed either pcDNA3.1-*GADD45A* or *GADD45A*-siRNA1# were injected into the right forebrains of BALB/c nude mice as previously described [37].

### Bioluminescence imaging

Tumor growth was monitored by BLI using the IVIS spectrum image system (Perkin, Elmer) as previously described [44]. Bioluminescence measurements were acquired on day 0, 7, and then weekly until the end of experiment. Animals were intraperitoneally injected with 150 mg/kg/10 mL D-luciferin (D-luciferin potassium salt 1G, PerkinElmer). Thirty minutes after luciferin administration, animals were anesthetized with 3% isoflurane and imaged in the IVIS imaging box. BLI was expressed as a total radiance in photons per sec/cm^2^ per steradian.

### Luciferase reporter assay

Luciferase reporter vectors were constructed by inserting the 3′-UTR of *GADD45A* downstream of the luciferase gene in the psiCHECK-2 vector (Promega, USA) at *Xho*I and *Not*I sites (TaKaRa). The 3′-UTR of *GADD45A* (containing the binding sites for miR-148a) was amplified from a U87 cDNA library with the following primers: forward: 5′-GCGCTCGAGGGCATCTGAATGAAAATAACTG-3′, and reverse: 5′-GATGCGGCCGCCCTGCATGGTTCTTTCTAA-3′. Five nucleotides in the miR-148 binding site were mutated in the 3′-UTR of *GADD45A*. The primer sequences for the mutated 3′-UTR were as follows: forward: 5′-GCGCTCGAG CTGAACCAAATTCGTGAGA-3′, and reverse: 5′-GATGCGGCCGC TTCCTGCATGGTTCTTTCTA-3′. HEK293 cells were co-transfected with the luciferase reporter systems and miR-148a mimics/miR-NC as indicated in the Figure legends. Luciferase activity was detected 48 h after transfection using the Dual Luciferase Reporter Assay System (Promega) following the manufacturer’s instructions. Data were normalized to Renilla activity.

### Cell migration and invasion assay

Transwell migration and invasion assays were performed using cell culture inserts in 24-well plates (BD Biosciences, San Jose, CA, USA). A 100 μl suspension containing 5×10^4^ cells from each subgroup in serum-free medium was added to the upper chamber. We added 0.6 ml medium containing 10% FBS to the lower chamber as a chemoattractant. For the migration assay, cells were incubated for another 20 h. For the invasion assay, we used Matrigel invasion chambers in 24-well plates (BD Biosciences). Cells were incubated for a further 42 h at 37°C in 5% CO_2_. Cells that had adhered to the lower well were fixed and stained with 0.1% crystal violet staining solution. The average number of stained cells was calculated from five different microscopic fields.

### Immunofluorescence staining

For immunofluorescence, cells were fixed in 4% paraformaldehyde and incubated in hydrogen peroxide to inhibit endogenous peroxidase activity. Non-specific antibody binding sites were blocked with 4% bovine serum albumin for 1 h and cells were labeled overnight with anti-β-catenin antibody (1:100 dilution; Abcam). After primary antibody incubation, cells were washed and labeled with the corresponding goat anti-mouse IgG (FITC) secondary antibody (1:1000 dilution; Abcam) for 2 h at 37°C. Cells were counterstained with DAPI and observed under a confocal microscope.

### Rescue experiments

To determine whether inhibiting GADD45A affected miR-148a-mediated effects, IDH1*^WT^* or IDH1*^R132H^* U87 cells were transfected with an miR-148a inhibitor and *GADD45A* siRNA1# or scr. After transfection, the expression of GADD45A and EMT markers were analyzed by immunoblotting. In addition, neurosphere formation was measured as described earlier [16].

### Statistical analysis

All results are presented as the mean ± standard deviation (SD) from three independent experiments performed in triplicate. Statistical analyses were performed using SPSS statistical software (Version 13.0; SPSS, Inc., Chicago, IL, USA). Survival curves were generated according to the Kaplan–Meier method and statistical analysis was performed using the Log-rank test. The Pearson Chi-square test was used to analyze the relationship between GADD45A expression and pathological features. The Student’s t-test or one-way ANOVA was used to analyze data from function analysis. P<0.05 was considered statistically significant.

## SUPPLEMENTARY FIGURE AND TABLE




