# Supplementary material for: MiR-148a increases glioma cell migration and invasion by downregulating GADD45A in human gliomas with IDH1 R132H mutations
Source: Oncotarget. 2017 Mar 3;8(15):25345–61. doi: 10.18632/oncotarget.15867 (PMC5421935; doi:10.18632/oncotarget.15867)
Supplement: Supplementary file 1 [file oncotarget-08-25345-s001.pdf]

# MiR-148a increases glioma cell migration and invasion by downregulating GADD45A in human gliomas with *IDH1* R132H mutations

## SUPPLEMENTARY FIGURE AND TABLE

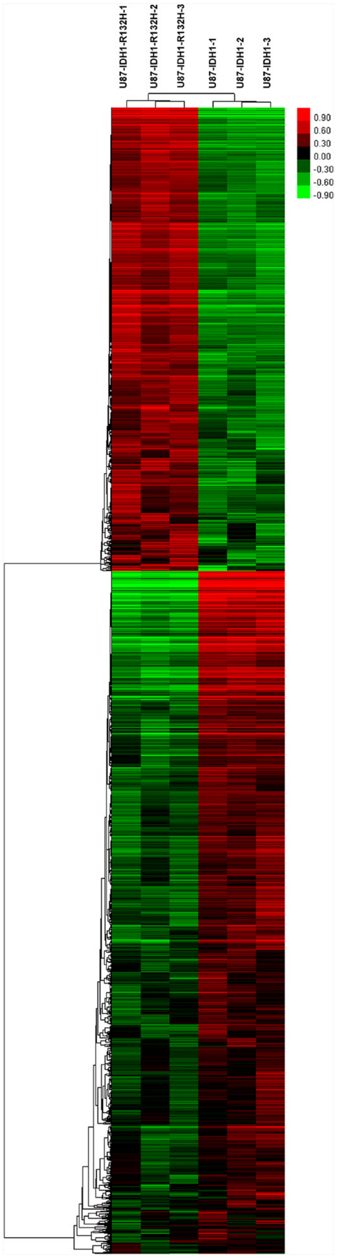


**Supplementary Figure 1: Microarray analysis of gene expression in IDH1$^{WT}$ and IDH1$^{R132H}$ glioblastoma cells.** *GADD45A* was among the differentially expressed genes and was downregulated in IDH1$^{R132H}$ gliomas compared with IDH1$^{WT}$ gliomas.

**Supplementary gene-list.xls:**

See Supplementary File 1
